# The Patients and the Hospitals

**Published:** 1913-03-01

**Authors:** 


### The Hospital

The Workers' Newspaper of Administrative Medicine and Institutional Life, Administration, National Insurance and Health.

No. 1391, Vol. LIII. SATURDAY, MARCH 1, 1913. PRICE ONE PENNY.

### THE PATIENTS AND THE HOSPITALS.

When the Insurance Act came into active working, so far as medical benefit is concerned, there was much doubt as to the effects it might have upon the number of patients treated at the voluntary hospitals. The managers of voluntary hospitals, naturally enough, laid it down as a principle that insured persons should not be dealt with in the out-patient departments of the hospitals when their ailments were such as could be treated by a practitioner of average ability. Such cases were to be handed on to the Insurance doctors. This was necessary, because, as insured persons, their treatment is the business of the private practitioner, who is to be paid by the Insurance Committees for supplying it. But it has been made manifest that those persons who have acquired the habit of attending at the hospitals prefer the treatment they obtain there to that of the panel practitioner. The right course to adopt in these cases may, therefore, soon become a pressing question for many of the voluntary hospitals. Are the voluntary hospitals to co-operate in helping the panel doctor to shirk his plain duty by admitting all applicants for out-patient relief, however simple their ailments, or are they to refuse to treat such patients in the out-patient department? It is perfectly clear that to co-operate with any panel doctor who shirks his plain duty will be to increase immeasurably hospital abuse, and to add greatly to the number of patients thus improperly admitted to hospital benefit. So this question is engaging the attention of hospital managers, especially where the number of patients is very large—a fact which points to the wisdom displayed by those hospitals which have determined to work the Insurance Act during the first few months with a slack rein, whilst they keep a careful record of all insured persons attending, and of the occurrence of such abuses and evils as those just mentioned. The difficulty to any other course arises from the fact that the voluntary hospitals have always made the interests of the patient the first consideration, whilst it is demonstrably provable that the working of the Insurance Act at present adds immeasurably to the hardships of many insured persons when ill, and fines them heavily by the loss of time and the delays attaching to treatment by a panel doctor.

The Insurance Act is, therefore, calculated to bring prominently to the front questions relating to the patient and the hospitals. As matters are proceeding at present, the voluntary hospital, as the one strong, impartially administered and sympathetic source of medical benefit, is placed between the devil and the deep sea. On the one hand it has brought constantly to its notice the cruelties and avoidable hardships to many poor people, when suffering from minor ailments, who may be cut off from treatment in the out-patient department of a hospital. On the other, it has to bear the burden of criticism and possible loss of income should it, as a matter of sympathy and good feeling to the poor, admit insured persons suffering from ailments which can and ought to be treated by practitioners of average ability. The latter course would, we fear, tend to increase largely the number of out-patients at the larger hospitals especially, with results which are not calculated to help these institutions financially. A further hardship for the hospitals is the attitude taken up by a certain type of persons, who in the past have done their best to show their gratitude to the hospitals by giving, say, a penny a week through the Hospital Saturday or some other artisans' fund. The view taken by these contributors was brought out clearly in The Hospital of February 22, in the article on the London Hospital Saturday Fund, where the Executive of that Fund report "a constant stream of complaints from the honorary collectors of the Fund," because the voluntary hospitals are trying to do their duty "by referring insured persons with minor ailments to the panel doctor." What the Executive ask is that the London hospitals shall grant days of grace to insured persons to realise the changed conditions and to arrange accordingly. This policy is being pursued by those hospitals where the slack-rein principle has been deliberately adopted for a fixed period.

Still, do what they will, the hospitals must accept what the Executive of the Saturday Fund describe as "indignation and ill-feeling" on the part of some patients, however just and proper their system of administration may, in fact, be. It is perfectly clear that the claims of the Hospital Saturday Executive that every patient, whether insured or not, shall have first treatment at the hospitals, and that "insured persons, even though they may not be serious cases, working many miles away from home, shall receive treatment, too, for the sake of humanity," cannot be accepted as a permanent arrangement. The blind-eye policy is often a wise one to pursue, as in the present instance, for a strictly limited period. It should enable the new order of things to settle down and become operative. In practice, however, the ultimate attitude of the hospitals in regard to out-patients must be to refuse to admit to hospital treatment those cases which can be treated properly by panel practitioners of average ability. So it may be gathered that the problem of the patient and the hospital was never of more importance than it is to-day.

